# Proposed Nomenclature for Landmarks in Anterior-Segment OCT: The APOSTEL-AS Panel Consensus

**DOI:** 10.1001/jamaophthalmol.2025.2414

**Published:** 2025-08-14

**Authors:** Alexander S. Fraser, Marcus Ang, Alice Bellchambers, Colin J. Chu, Alastair K. Denniston, Laura E. Downie, Thomas Evans, Scott Hau, Alex S. Huang, Pearse A. Keane, Xiaoxuan Liu, Jodhbir S. Mehta, Giovanni Ometto, Axel Petzold, Edmund Tsui, Tamara S. Fraser, Benjamin Xu, Caroline Thaung, Ameenat L. Solebo

**Affiliations:** 1 Population, Policy and Practice Department, University College London, Great Ormond Street Institute of Child Health, London, United Kingdom; 2 Trinidad Eye Hospital, San Fernando, Trinidad and Tobago; 3 Southend Eye Hospital, Essex, United Kingdom; 4 Cornea and External Eye Disease Service and Refractive Service, Singapore National Eye Center, Singapore; 5 DUKE-NUS Department of Ophthalmology and Visual Sciences, Singapore; 6 University Hospitals Sussex NHS Foundation Trust, United Kingdom; 7 Moorfields NIHR Biomedical Research Centre, London, United Kingdom; 8 University College London Institute of Ophthalmology, London, United Kingdom; 9 NIHR Birmingham Biomedical Research Centre, Birmingham, United Kingdom; 10 College of Medicine and Health, University of Birmingham, Birmingham, United Kingdom; 11 Department of Optometry and Vision Sciences, The University of Melbourne, Parkville, Victoria, Australia; 12 Department of Ophthalmology, Sheffield Teaching Hospitals NHS Foundation Trust, Sheffield, United Kingdom; 13 Hamilton Glaucoma Center, The Viterbi Family Department of Ophthalmology, Shiley Eye Institute, University of California, San Diego; 14 Birmingham Health Partners Centre for Regulatory Science and Innovation, Birmingham, United Kingdom; 15 Singapore Eye Research Institute, Singapore; 16 University College London, University College London Institute of Neurology, London, United Kingdom; 17 City St George’s, University of London, Department of Optometry & Visual Sciences, London, United Kingdom; 18 Ocular Inflammatory Disease Center, UCLA Stein Eye Institute, David Geffen School of Medicine at UCLA, Los Angeles, California; 19 Mid South Essex NHS Foundation Trust, Essex, United Kingdom; 20 USC Roski Eye Institute, Keck School of Medicine at USC, Los Angeles, California; 21 NIHR Great Ormond Street Biomedical Research Centre, London, United Kingdom; 22 Ophthalmology Department, Great Ormond Street Hospital, London, United Kingdom

## Abstract

**Importance:**

Anterior-segment optical coherence tomography (AS-OCT) has broad clinical and research utility. The utility of quantitative data derived from AS-OCT is, however, dependent on the quality and consistency of the cumulative evidence base.

**Objective:**

To develop consensus-based nomenclature that supports standardized reporting of AS-OCT image acquisition and analyses as a foundation to improve research reproducibility.

**Design, Setting, and Participants:**

A multistage consensus exercise was undertaken, in 2024, with an expert panel of ophthalmologists, optometrists, and vision scientists, informed by a scoping review to identify the range of AS-OCT applications and terminologies in use. Panel members were selected to represent the breadth of clinical areas identified within the review or key stakeholder groups (ie, ocular image acquisition, imaging analysis, consensus methodologies). Nominal group technique was used to seek consensus on existing and newly proposed terminologies for normal anatomical structures identifiable within cross-sectional swept-source and spectral-domain AS-OCT images. An ophthalmic histopathologist then reviewed the output for concordance with histological terminology. Data were analyzed from July 2024 to January 2025.

**Exposures:**

Expert consensus on nomenclature of ocular structures.

**Main Outcomes and Measures:**

Agreement on nomenclature of ocular structures, with consensus defined as at least 80% expert agreement.

**Results:**

A multinational group of 14 experts (mean [SD] age, 46.1 [7.8] years; 10 male [71%]) participated in the consensus process. Scoping review findings resulted in the generation of 45 terms for the annotation of identifiable structures. After presentation of multiple image annotations representing these structures, consensus was reached for 31 terms across 7 images. There was consensus on the use of standardized descriptive terms from the Federative Committee on Anatomical Terminology (FCAT) alongside established eponymous terms. There was absence of consensus on annotations and visualization of structures within less optically accessible areas, such as the episcleral and scleral vasculature.

**Conclusions and Relevance:**

Results of this survey study describe international, multidisciplinary consensus on terminology for ocular structures identifiable in AS-OCT images and present representative annotated images for reference use. Future expansion of nomenclature and annotation are anticipated, facilitated by advances in imaging technology. Follow-up consensus exercises should consider consensus-based terminology and annotations of pathological structural changes.

## Key Points

**Question:**

Can consensus terminology for anterior-segment ocular structures identifiable on cross-sectional optical coherence tomography be developed?

**Findings:**

In this survey study using a nominal group technique, a panel comprising 14 eye care clinician/researcher experts reached consensus (at least 80% agreement) on nomenclature for ocular structures identifiable on cross-sectional anterior-segment optical coherence tomography images.

**Meaning:**

Results suggest that consensus nomenclature for normal anterior-segment structures identifiable on anterior-segment optical coherence tomography could be used to standardize reporting in studies to advance interoperability and replicability of future work.

## Teaser

This survey study investigates if consensus terminology can be developed for anterior-segment ocular structures identifiable on cross-sectional optical coherence tomography.

## Introduction

The advent of anterior-segment optical coherence tomography (AS-OCT) has transformed the visualization and assessment of the ocular AS.^1^ AS-OCT provides high-definition, cross-sectional images that allow for detailed analysis of both normal and pathological anatomy. This imaging modality has been used to describe health and disease states in a diverse group of disorders affecting structures including the ocular adnexa and surface,^2^ tear film,^3^ cornea,^4,5^ anterior chamber,^3,6,7^ iris,^8^ lens,^9^ and anterior chamber angle.^10,11,12,13^ However, as the use of this technology expands in both research and clinical practice, the lack of standardization of AS-OCT anatomical terminology is a major drawback, limiting the ability to compare findings across studies.^14^

Establishing a consensus-based nomenclature for the ocular structures imaged by AS-OCT has the capacity to advance scientific research and clinical care. Standardized annotation should enhance the accuracy and reproducibility of studies, facilitate collaboration across disciplines, and improve the communication of findings between clinicians and researchers. Such standardization (for annotation of retinal structures visible on posterior-segment OCT) was the outcome of the earlier consensus nomenclature presented by the original Advised Protocol for OCT Study Terminology and Elements (APOSTEL) group.^15^ The wider APOSTEL checklist also provides consensus recommendations for the reporting of quantitative posterior-segment OCT study results.^15,16^ An extension for APOSTEL, which ensures appropriateness and applicability of checklist recommendations for AS images (APOSTEL-AS), is currently under way.^17^

This article aims to address the need for standardized nomenclature for ocular structures visible on AS-OCT, by bringing together a group of experts from ophthalmology, optometry, and vision science to develop and describe consensus around terminology. Such nomenclature could be of benefit to a global audience of eye health professionals and researchers.

## Methods

This multistage study was developed using the Accurate Consensus Reporting Document, or ACCORD recommendations.^18^ ACCORD is the EQUATOR (Enhancing the Quality and Transparency of Health Research) Network–adopted guideline for reporting consensus-based methods in biomedical research and clinical practice. This work is part of the wider APOSTEL-AS, which has been registered with the EQUATOR network.^19^ A consensus-based method was selected to ensure international, multidisciplinary representation. Ethics approval for this work was granted by the University College London Institutional Review Board.

### Stage 1: Scoping Review

Item generation for the consensus exercise was informed by a scoping review. This review aimed to describe the use of AS-OCT across a range of disease areas and thus inform the selection of anatomical landmark annotations for the consensus exercise. A literature search was performed using a strategy with keywords and Medical Subject Headings (MESH) terms relating to AS-OCT (search methods detailed in the eAppendix in Supplement 1) to identify eligible studies. Studies were included if they described primary research findings that included quantitative outputs from AS-OCT imaging of living human eyes. At least 2 of the reviewers (A.S.F., A.B., T.S.F., A.L.S.) screened each citation’s title and abstract for eligibility. Information on disease or anatomical areas studied and country of origin for the research were extracted from studies deemed eligible for inclusion.

### Stage 2: Panel Selection

The consensus panel was formed through approach by the panel chair (A.L.S.) or by a snowball approach through other panel members. Data were gathered on panel participant age, gender, and country of practice. Panel member selection was based on demonstrable expertise in areas considered to hold high applicability for AS-OCT primary research, or for the consensus process. An operations committee (A.L.S., A.B., T.S.F., and A.S.F.) comprising the chair and early-career ophthalmologists was also formed to support the process by undertaking the scoping review and analysis of extracted data.

### Stage 3: Annotation Preparation

From the review of included studies, the operations committee collated a selection of cross-sectional AS-OCT images with initial labeling suggestions based on normal anatomical landmarks identified as areas of subject interest. Anonymized images were used with written participant consent for the use of nonidentifiable images in research. Participants did not receive compensation or incentives to participate.

### Stage 4: Assessing Consensus

A nominal group method was used to reach consensus. Terminologies were presented to the panel as annotations on cross-sectional AS-OCT images. These annotations were distributed 2 weeks before a virtual discussion meeting to allow panel members to consider the appropriateness of each presented term for the annotated ocular structures within each image. At the virtual meeting, the panel chair facilitated the sharing of panel member judgments on labels, ensuring open discussion to identify plurality of judgment on appropriate terms for ocular structure or the applicability of terms.

Panel members then voted on each term (anonymity was not used) with the threshold for consensus set at 80% agreement across members. Details of agreement, any abstention, and any reasons for failure to reach the consensus threshold were recorded. After confirmation of consensus, an ophthalmic histopathologist reviewed each annotation for concordance with histological terminology. No financial incentives were used to encourage participation among this consensus group. Responses were collected using a REDCap (Vanderbilt University) form developed by and piloted within the operations committee.

### Statistical Analysis

Descriptive statistics were used to summarize the collected data. Categorical variables were reported as counts and percentages. Where applicable, continuous variables were summarized using mean or median, SD, and range. All data were analyzed using Microsoft Excel. The analysis was conducted between July 2024 to January 2025.

## Results

### Scoping Review

A multinational group of 14 experts (mean [SD] age, 46.1 [7.8] years; 4 female [29%]; 10 male [71%]) participated in the consensus process. The database search identified 4765 articles, of which 3214 articles were eligible for inclusion. Across these articles, there was representation of different clinical areas: 1400 (43%) concerned corneal anatomy or pathology, 1024 (32%) glaucoma, 810 (25%) cataract or the crystalline lens, 130 (4%) uveitis, 119 (4%) tear film, and 59 (2%) strabismus or extraocular muscles.

With regard to the country in which the research originated, 1535 articles (48%) were from teams based within the US, followed by 639 (20%) from the UK, 165 (5%) from Switzerland, and 175 (5%) from India. Representation from other regions was minimal by comparison but mostly comprised China, Japan, New Zealand, and other European countries (ie, Germany, France, Netherlands). Stratified by economic setting (using the Organisation for Economic Co-operation and Development 2023 database),^20^ 2907 articles (90%) came from high-income countries, with 190 (6%) from lower-middle–income countries, 115 (4%) from upper-middle–income countries, and 2 (<1%) from low-income countries.

### Panel Composition

The resultant consensus panel had expertise in glaucoma (A.S.H., B.X., P.A.K., T.S.F.), cornea (M.A., S.H., J.S.M.), ocular surface disease (L.E.D., S.H.), uveitis (C.J.C., E.T., X.L., A.L.S), consensus methodology (A.K.D., X.L., A.L.S.), imaging acquisition (T.E., A.L.S.), ophthalmic imaging data science (G.O., P.A.K.), and also representation of membership of the original APOSTEL Posterior-Segment OCT Nomenclature Consensus Working Group (A.P.).

### Annotation Preparation

The operation committee identified 45 terms for ocular structures used for the generation of quantitative data from AS-OCT images. Seven anonymized cross-sectional images were selected for annotation with the identified terms. These annotated images, and a list of terms, are available as a supplementary document (eFigures 1-6 and eTable 1 in Supplement 1, respectively).

### Consensus Exercise

**Figure 1.**
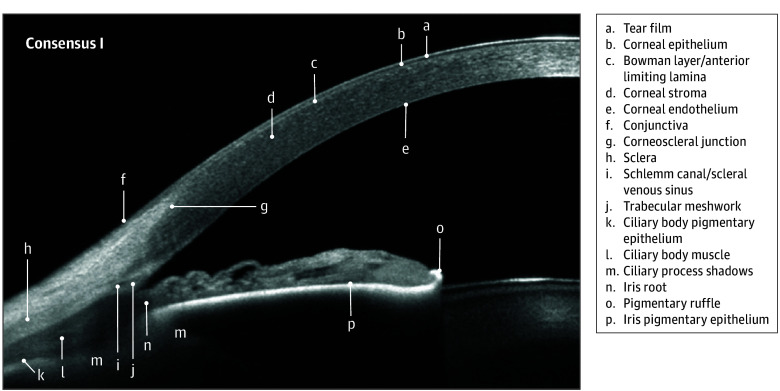
Advised Protocol for OCT Study Terminology and Elements Extension for Anterior Segment (APOSTEL-AS) Consensus Nomenclature I: AS Overview Nomenclature for normal anatomic landmarks seen on swept-source optical coherence tomography (OCT) images adopted by the APOSTEL-AS panel. The anterior chamber of a 35- to 45-year-old female was imaged using the CASIA2 OCT (Tomey).

**Figure 2.**
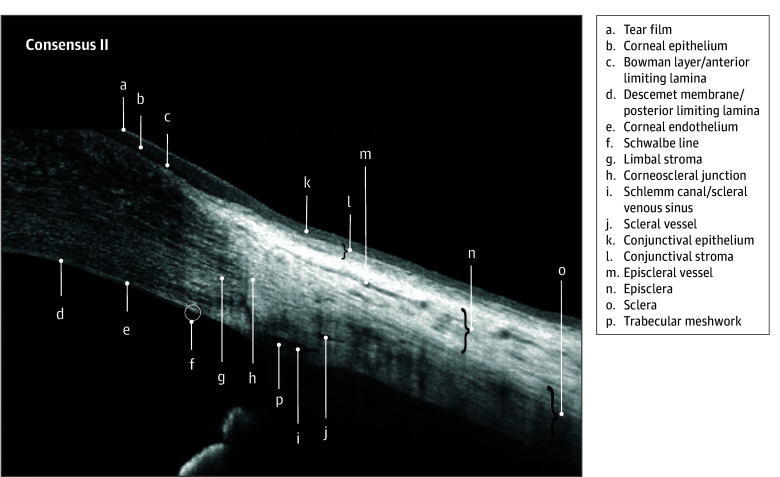
Advised Protocol for OCT Study Terminology and Elements Extension for Anterior Segment (APOSTEL-AS) Consensus Nomenclature II: Corneoscleral Annotations Nomenclature for normal anatomic landmarks seen on spectral-domain optical coherence tomography (OCT) images adopted by the APOSTEL-AS panel. The anterior chamber of a 15- to 25-year old female was imaged using the Avanti OCT (Optovue).

**Figure 3.**
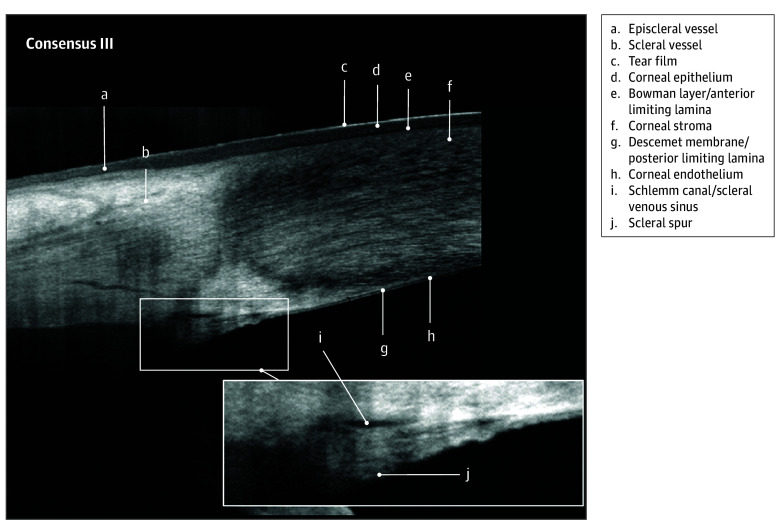
Advised Protocol for OCT Study Terminology and Elements Extension for Anterior Segment (APOSTEL-AS) Consensus Nomenclature III: Limbal and Anterior Chamber Angle Annotations Nomenclature for normal anatomic landmarks seen on spectral-domain optical coherence tomography (OCT) images adopted by the APOSTEL-AS panel. The healthy anterior chamber of a 15- to 25-year-old male was imaged using the Avanti OCT (Optovue).

**Figure 4.**
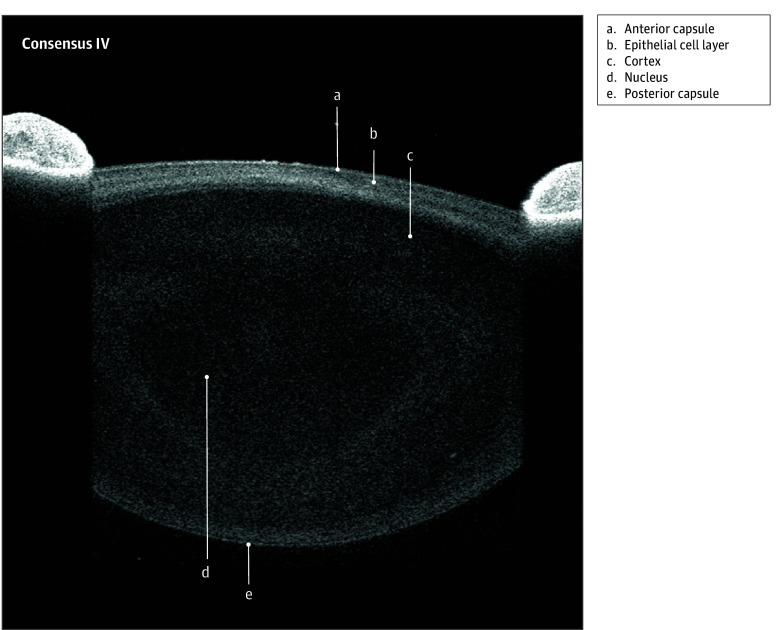
Advised Protocol for OCT Study Terminology and Elements Extension for Anterior Segment (APOSTEL-AS) Consensus Nomenclature IV: Lens Annotations Nomenclature for normal anatomic landmarks seen on swept-source optical coherence tomography (OCT) images adopted by the APOSTEL-AS panel. The healthy crystalline lens of a 15- to 25-year-old female was imaged using the Anterion OCT (Heidelberg).

All 14 invited panel members participated in the nominal group technique activity. The nomenclature that reached the consensus threshold comprised 31 of the 45 terms across 7 images. These terms are listed in the Table and identified in Figure 1, Figure 2, Figure 3, and Figure 4

**Table.** Advised Protocol for OCT Study Terminology and Elements Extension for Anterior Segment (APOSTEL-AS) Consensus AS–Optical Coherence Tomography (OCT) Nomenclature^a^

Cornea/tear film	Conjunctiva	Episclera/sclera	Limbus	Iris/ciliary body	Lens
Bowman layer/anterior limiting lamina (Figure 1, Figure 2, and Figure 3)	Conjunctiva (Figure 1)	Episclera (Figure 2)	Corneoscleral junction (Figure 1 and Figure 2)	Ciliary body muscle (Figure 1)^b^	Anterior capsule (Figure 3)
Corneal endothelium (Figure 1, Figure 2, and Figure 3)	Conjunctival epithelium (Figure 2)	Episcleral vessel (Figure 2 and Figure 3)^b^	Limbal stroma (Figure 2)	Ciliary body pigmentary epithelium (Figure 1)	Cortex (Figure 3)
Corneal epithelium (Figure 1, Figure 2, and Figure 3)	Conjunctival stroma (Figure 2)	Sclera (Figure 1 and Figure 2)	Schlemm canal/scleral venous sinus (Figure 1, Figure 2, and Figure 3)	Ciliary process shadows (Figure 1)	Epithelial cell layer (Figure 3)
Corneal stroma (Figure 1, Figure 2, and Figure 3)	NA	Scleral spur (Figure 3)	Schwalbe line (Figure 2)	Iris pigmentary epithelium (Figure 1)	Nucleus (Figure 3)
Descemet membrane/posterior limiting lamina (Figure 2 and Figure 3)	NA	Scleral vessel (Figure 2 and Figure 3)	Trabecular meshwork (Figure 1 and Figure 2)	Iris root (Figure 1)	Posterior capsule (Figure 3)
Tear film (Figure 1, Figure 2, and Figure 3)	NA	Insertion rectus muscle tendon	NA	Pigmentary ruffle (Figure 1)	NA

Abbreviation: NA, not applicable.

^a^ Consensus terms describing AS-OCT structures that achieved consensus (minimum 80%) expert panel agreement organized by their anatomical distribution.

^b^ One term did not reach unanimous agreement.

### Terminology

The use of Federative Committee for Anatomical Terminology (FCAT; such as *anterior limiting lamina*, *posterior limiting lamina*, and *scleral venous sinus*)^21^ rather than eponymous terms (such as *Bowman layer*, *Descemet membrane*, and *canal of Schlemm*, respectively) was suggested during group discussion. Consensus could not be reached on the use of only eponymous terms (high community adoption)^22^ or the use of only FCAT terms (internationally accepted but with relatively low current diffusion across the clinical community). Consensus was reached with the balance of using both terminologies within the APOSTEL-AS nomenclature.

Terminology was also changed (through consensus) where initial nomenclature claimed higher specificity than imaging could support. For example, where *ciliary body radial fibers* could not be distinguished from circular or longitudinal fibers, there was consensus to rename this annotation to *ciliary body muscle*. Similarly, a *scleral plexus* and *episcleral plexus* were renamed *scleral vessels* and *episcleral vessels*, respectively, as the type or caliber of lumen could not be specified.

Of the 31 consensus terms reviewed, 30 achieved unanimous agreement among the panel. The only exception was the term *ciliary body muscle*, which achieved a high level of consensus (88.9%).

### Annotations Not Reaching Consensus for Inclusion

There was unanimity on the removal of the BELL, or band of extracanalicular limbal lamina, Tenon capsule, and sphincter pupillae annotations (eFigures 1-6 in Supplement 1). High consensus (80% to <100% but not reaching unanimity) was reached for removing labels and nomenclature associated with *Schwalbe line* (83%) and *palisades of Vogt* (90%). The most common reason for failure to reach consensus on annotations was disagreement on whether an anatomical microstructure could be sufficiently clearly located or repeatably delineated. In the case of 2 annotations, the nominal group discussion agreed that although consensus was not reached for the presented label, it might be possible to delineate structures using cross-sectional AS swept-source and spectral-domain OCT images of health and disease states. These structures comprised the *anterior hyaloid*, which members postulated might be delineated from the retrolental space with postacquisition OCT image processing, and (lenticular) *epicortex*, which might be identifiable in cases with lens opacities.

## Discussion

From this multidisciplinary activity, we report international consensus on recommended terminology for physiological ocular structures identifiable using AS-OCT and share representative annotated images suitable for reference use. The most common obstacle to achieving consensus was the limited ability of current AS-OCT imaging platforms to locate and delineate ocular structures. The use of both the eponymous and FCAT terminology was agreed on in order to support anatomical accuracy and practical adoption. Unanimity was reached for almost all annotations across both swept-source and spectral-domain OCT images, supporting applicability of this nomenclature across a diverse group of clinicians and researchers.

The development of consensus-based nomenclature for AS structures visible on AS-OCT represents an important step in the standardization of ocular imaging.^23^ Similar standardization for posterior-segment OCT image nomenclature was a key foundation for the subsequent work on identifying ocular biomarkers in both ocular and nonocular disease.^3,24,25^ By establishing a unified language for describing AS structures, this APOSTEL-AS initiative has addressed a gap that might hamper communication across the research and clinical communities.^26^ The consensus nomenclature may also enhance diagnostic precision, facilitate comparative research, and support AS-OCT derived quantitative data. This is particularly important in an era of increasingly personalized medicine,^25,26,27^ where precise imaging and diagnostics are essential for tailoring care to individual patient needs.^28^

Consensus on annotations was not achieved across all ocular structures initially considered. This likely reflects the inaccessibility of some of these structures to direct clinical in vivo visualization. Anatomical features such as the palisades of Vogt, or scleral and episcleral vasculature, are typically identified on histological sections. However, their corresponding OCT appearances have not yet been confidently defined. Additionally, the OCT hyperreflectivity signals from deeper bodies surrounded by tissues of similar reflectivity results in less distinct delineation. A possible example of this is the band of extracanalicular limbal lamina. These deeper structures also provide less apparent signals when compared with those from superficial structures less impacted by OCT light scattering. These factors are likely to result in some anatomical landmarks being less familiar to clinical researchers, leading to a failure to reach consensus on annotations. The lack of consensus on these annotations does not preclude the future capture of these structures with newer or modified OCT platforms.

The initial APOSTEL work drew on a preceding posterior-segment nomenclature study conducted by the International Nomenclature for Optical Coherence Tomography (IN-OCT) panel.^29^ IN-OCT and APOSTEL-AS share the aim to establish consensus-based standardized nomenclature for OCT-derived anatomical landmarks, the involvement of selected panel members with relevant expertise, systematic selection of proposed nomenclature, the use of annotated OCT images to support a consensus approach, and structured decision-making processes. Both ensure transparency by documenting levels of agreement and contentious areas and supporting future extension of the work. The methods used differ, however, in their frameworks and execution. The approach used by the IN-OCT panel was pragmatic and efficient, relying on expert judgment and iterative consensus. In contrast, the APOSTEL-AS panel benefited from a structured and validated framework but required more time and resources due to its multistage process and documentation. Specifically, preparation processes diverged, with IN-OCT annotations developed through expert opinion, whereas the APOSTEL-AS annotations were underpinned by a scoping review, to ensure comprehensive coverage of potential ocular structures. The IN-OCT investigators adopted an ad hoc approach, with discussion and negotiation undertaken until a unanimous consensus name was adopted for annotations. APOSTEL-AS consensus methods were consistent with the ACCORD recommendations,^18^ with more structured, reproducible processes, and with documentation and quantification of the degree of absence of agreement, highlighting areas in need of future work with regard to AS-OCT visualization. Overall, the systematic approach of the APOSTEL-AS panel should better support its utility and adoption across a wider, multidisciplinary ophthalmic community. This community includes those working on the sclera and episclera, where improved visualization of structure and vasculature might hold benefit for strabismus surgical planning or the monitoring of inflammatory disorders; corneal disease, where improved visualization and differentiation of limbal palisades might hold biomarkers for degenerative disease; or glaucoma, through improved understanding of aqueous outflow structures. It is possible that these structures are more easily identifiable on AS-OCT images of eyes with established pathology.^30^ However, it is imperative that these nomenclatures are applicable in the absence of pathological features. Our improved understanding of the power of population imaging epidemiology^31^ and the normative states of ocular structure are key to the development of imaging biomarkers able to predict and, therefore, support the prevention of clinically meaningful negative outcomes.

### Future Directions

Advancements in OCT technology, and the development of other imaging technologies, are expected to improve the visualization of AS structures. These advancements include deconvolution in attaining artifact-free OCT images,^32^ adaptive optics,^33^ polarization sensitivity,^34^ and ultra-high resolution^35^ alongside functional OCT approaches such as ocular coherence elastography,^36^ OCT Doppler angiography,^37^ and functional in vivo confocal microscopy.^38^ Further developments in AS-OCT analytical software are also expected to change how we view the AS, providing unprecedented views of complex structures. Such work may, eg, aid descriptions of anatomical landmarks contextualized by ocular location. For example, the differing relative hyperreflectivity of Bowman layer in the central cornea vs the limbus is likely to reflect the differing thickness of Bowman layer across the cornea^39^ and/or any differences in the overlying or underlying structures. Another example might be improved differentiation of Descemet membrane and corneal endothelium in premorbid states. This document provides a foundation for those expected future developments.

The utility of any consensus recommendation is dependent on the adoption of that guidance by the community toward whom it is directed. The target audience for the use of these foundational APOSTEL-AS nomenclature reference tools (ie, the annotated images and associated terminology) is the clinical and biomedical research community who work with quantitative and qualitative data derived from AS ocular AS-OCT. The integration of this work with the APOSTEL-AS panel, an EQUATOR reporting guideline,^19^ will support dissemination and future adoption.

### Limitations

A consensus-based survey study has inherent limitations. Obtaining agreement among diverse groups of experts can be a slow, iterative process. There is a risk of oversimplifying complex anatomical features in the pursuit of standardization. Also, attempts to create a “best of both worlds” nomenclature that suits both clinical and research purposes can lead to potential discrepancies in how specific structures are represented. The involvement of an ocular histopathologist in this consensus work should support the acceptability of the terms which reached consensus, irrespective of the identity of the audience.

Although this consensus represents the current state of knowledge, it should be adaptable to accommodate future innovations, discoveries or amendments in nomenclature. This adaptability, and the applicability of this work, is supported by the use of spectral-domain and swept-source OCT imaged within the consensus work, with the different specifications of these machines (eTable 2 in Supplement 1) likely to have an impact on the output image. Future work should address gaps such as the absence within the FCAT of anatomically descriptive nomenclature for eponymously named structures such as Schwalbe line and descriptors of ocular structures, which respect any differences in visualization across systems or across ocular locations.^21^

An additional shortcoming of this study is the lack of articles, or panel members, originating from lower-income countries, where the burden of disease of the AS (particularly cataract and corneal disease) is high.^40^ Therefore, subject areas of relevance may not have been explored. However, there is diversity in the international representation of the review and consensus activity.

## Conclusions

In this survey study, the establishment of a consensus-based nomenclature for AS-OCT structures marks a crucial milestone in the field of AS imaging. Although challenges remain in ensuring their flexibility and adaptation to future discoveries, the benefits of improved communication, research reproducibility, and clinical consistency should be clear. Continued collaboration and refinement will be key to ensuring that this standardized nomenclature remains a dynamic and useful tool for advancing ocular health care and research.

## Notes

Supplement 1. **eAppendix.** Literature Search Strategy **eTable 1.** Terms Presented to Consensus Group **eFigure 1.** Anterior Chamber Labels Presented to Consensus Group **eFigure 2.** Corneal Labels Presented to Consensus Group **eFigure 3.** Limbal and Corneoscleral Labels Presented to Consensus Group **eFigure 4.** Limbal and Corneoscleral Labels Presented to Consensus Group, Part 2 **eFigure 5.** Limbal and Anterior Chamber Angle Labels Presented to Consensus Group **eFigure 6.** Crystalline Lens Structure Labels Presented to Consensus Group **eTable 2.** Details of Imaging Systems Used

Supplement 2.
Data Sharing Statement.
