# Supplementary material for: Proposed Nomenclature for Landmarks in Anterior-Segment OCT: The APOSTEL-AS Panel Consensus
Source: JAMA Ophthalmol. 2025 Aug 14;143(9):749–57. doi: 10.1001/jamaophthalmol.2025.2414 (PMC12355391; doi:10.1001/jamaophthalmol.2025.2414)
Supplement: Supplement 1. — eAppendix. Literature Search Strategy eTable 1. Terms Presented to Consensus Group eFigure 1. Anterior Chamber Labels Presented to Consensus Group eFigure 2. Corneal Labels Presented to Consensus Group eFigure 3. Limbal and Corneoscleral Labels Presented to Consensus Group eFigure 4. Limbal and Corneoscleral Labels Presented to Consensus Group, Part 2 eFigure 5. Limbal and Anterior Chamber Angle Labels Presented to Consensus Group eFigure 6. Crystalline Lens Structure Labels Presented to Consensus Group eTable 2. Details of Imaging Systems Used [file jamaophthalmol-e252414-s001.pdf]

Supplementary Online Content

Fraser AS, Ang M, Bellchambers A, et al. Proposed nomenclature for landmarks in anterior-segment OCT: the APOSTEL-AS panel consensus. *JAMA Ophthalmol*. Published online August 14, 2025. doi:10.1001/jamaophthalmol.2025.2414

eAppendix. Literature Search Strategy

eTable 1. Terms Presented to Consensus Group

eFigure 1. Anterior Chamber Labels Presented to Consensus Group

eFigure 2. Corneal Labels Presented to Consensus Group

eFigure 3. Limbal and Corneoscleral Labels Presented to Consensus Group

eFigure 4. Limbal and Corneoscleral Labels Presented to Consensus Group, Part 2

eFigure 5. Limbal and Anterior Chamber Angle Labels Presented to Consensus Group

eFigure 6. Crystalline Lens Structure Labels Presented to Consensus Group

eTable 2. Details of Imaging Systems Used

This supplementary material has been provided by the authors to give readers additional information about their work.

© 2025 Fraser AS et al. *JAMA Ophthalmology*.

eAppendix. Literature search strategy

Medline searched from database inception to 24th June 2024, using these search terms

#1	**anterior eye segments**<br>"anterior eye segment"[MeSH Terms] OR ("anterior"[All Fields] AND "eye"[All Fields] AND "segment"[All Fields]) OR "anterior eye segment"[All Fields]
#2	**optical coherence tomography**<br>"tomography, optical coherence"[MeSH Terms] OR ("tomography"[All Fields] AND "optical"[All Fields] AND "coherence"[All Fields]) OR "optical coherence tomography"[All Fields] OR ("optical"[All Fields] AND "coherence"[All Fields] AND "tomography"[All Fields])
#3	#1 AND #2
#4	**anterior segment optical coherence**<br>("anterior"[All Fields] OR "anteriores"[All Fields] OR "anteriorization"[All Fields] OR "anteriorized"[All Fields] OR "anteriors"[All Fields]) AND ("segment"[All Fields] OR "segment s"[All Fields] OR "segmental"[All Fields] OR "segmentally"[All Fields] OR "segmentals"[All Fields] OR "segmentation"[All Fields] OR "segmentational"[All Fields] OR "segmentations"[All Fields] OR "segmented"[All Fields] OR "segmenter"[All Fields] OR "segmenters"[All Fields] OR "segmenting"[All Fields] OR "segments"[All Fields]) AND ("eye"[MeSH Terms] OR "eye"[All Fields] OR "optic"[All Fields] OR "optic s"[All Fields] OR "optical"[All Fields] OR "optically"[All Fields] OR "optics"[All Fields]) AND ("coherence"[All Fields] OR "coherences"[All Fields] OR "coherencies"[All Fields] OR "coherency"[All Fields] OR "coherent"[All Fields] OR "coherently"[All Fields])
#5	**anterior segment oct**<br>("anterior"[All Fields] OR "anteriores"[All Fields] OR "anteriorization"[All Fields] OR "anteriorized"[All Fields] OR "anteriors"[All Fields]) AND ("segment"[All Fields] OR "segment s"[All Fields] OR "segmental"[All Fields] OR "segmentally"[All Fields] OR "segmentals"[All Fields] OR "segmentation"[All Fields] OR "segmentational"[All Fields] OR "segmentations"[All Fields] OR "segmented"[All Fields] OR "segmenter"[All Fields] OR "segmenters"[All Fields] OR "segmenting"[All Fields] OR "segments"[All Fields]) AND "oct"[All Fields]
#6	#3 OR #4 OR #5

© 2025 Fraser AS et al. *JAMA Ophthalmology*.

eTable 1. Terms presented to consensus group

Terms which reached consensus for a label are indicated in **bold**.

**Anterior capsule**	**Corneal stroma**	**Posterior capsule**
Anterior hyaloid space	**Corneoscleral junction**	Pupillary sphincter
Aqueous vein	**Cortex**	Schlemm's canal
Band of extracanalicular limbal lamina (BELL)	**Descemet's membrane**	**Schlemm's canal / scleral venous sinus**
**Bowman's layer**	Dilator pupillae	**Schwalbe's line**
Bowman's membrane	**Episclera**	**Sclera**
Bowman's membrane / Anterior limiting lamina	Episcleral plexus	Scleral plexus
**Ciliary body muscle**	**Episcleral vessel**	**Scleral spur**
**Ciliary body pigmentary epithelium**	**Epithelial cell layer**	**Scleral vessel**
Ciliary body radial fibres	**Insertion rectus muscle tendon**	**Tear film**
**Ciliary process shadows**	**Iris pigmentary epithelium**	Tear film hyper-reflexivity
**Conjunctiva**	**Iris root**	Tenon's capsule
**Conjunctival epithelium**	**Limbal stroma**	**Trabecular meshwork**
**Conjunctival stroma**	Limbus palisade of Vogt	
**Corneal endothelium**	**Nucleus**	
**Corneal epithelium**	**Pigmentary ruffle**	

© 2025 Fraser AS et al. *JAMA Ophthalmology*.

eFigure 1. Anterior chamber labels presented to consensus group.

Swept source OCT of 35-45 year old female acquired using Tomey CASIA2.

eFigure 1A. Annotated Image.

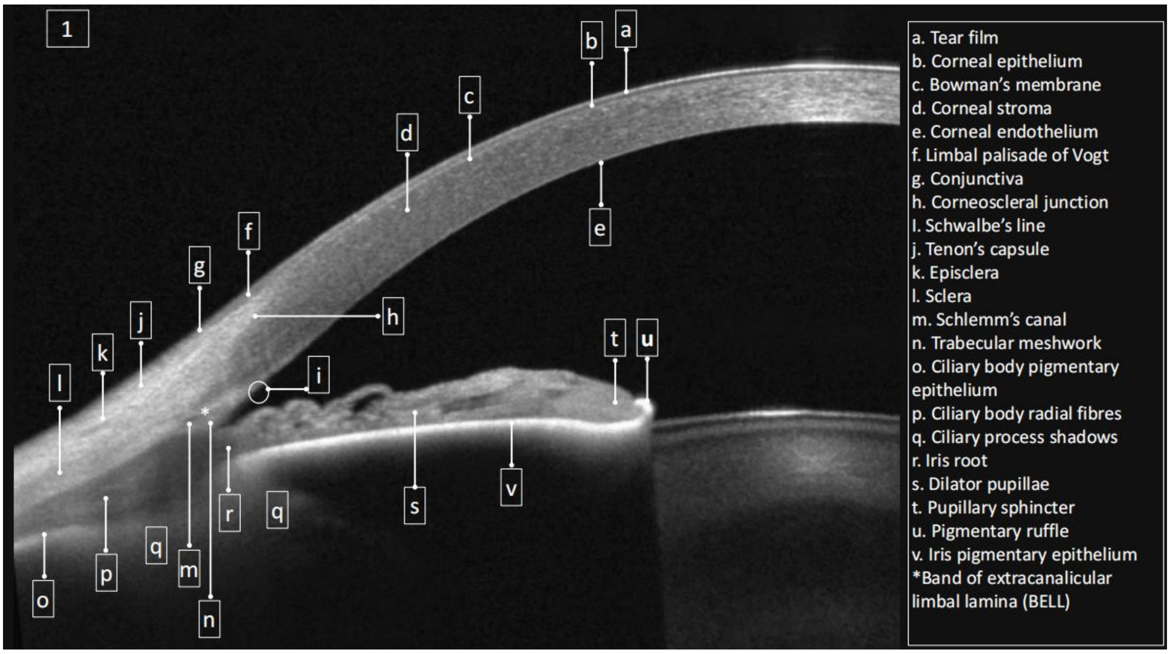

eFigure 1B. Unannotated Image

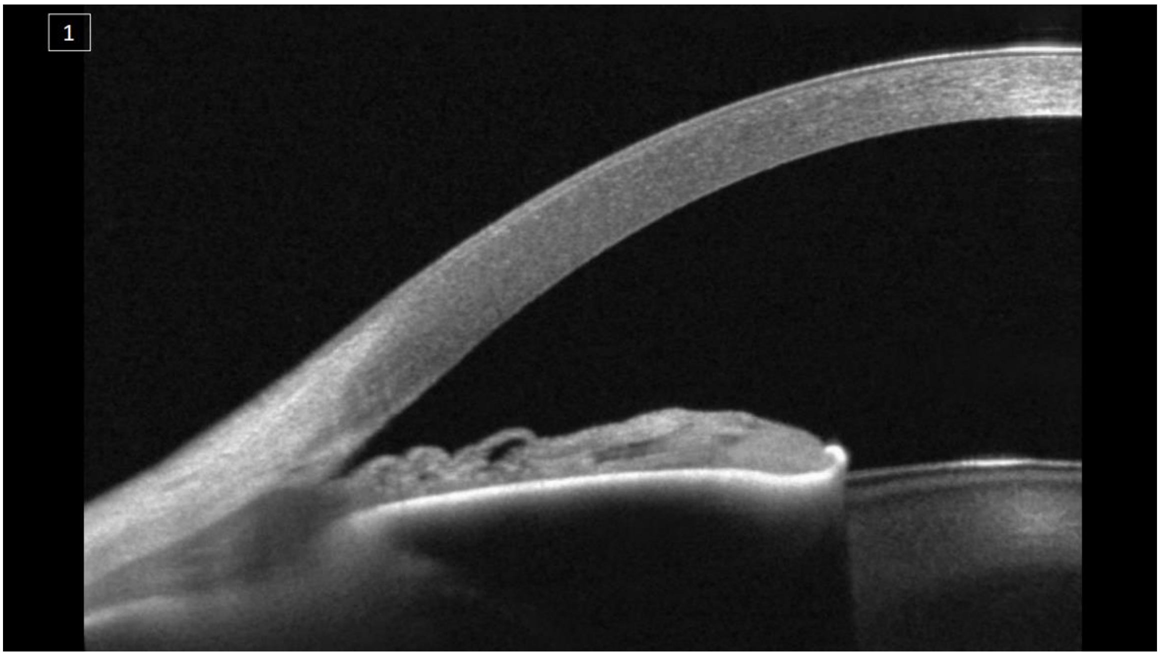

© 2025 Fraser AS et al. *JAMA Ophthalmology*.

eFigure 2. Corneal labels presented to consensus group.

Spectral domain OCT of 45-55 year old female acquired using Optovue Avanti

eFigure 2A. Annotated Image

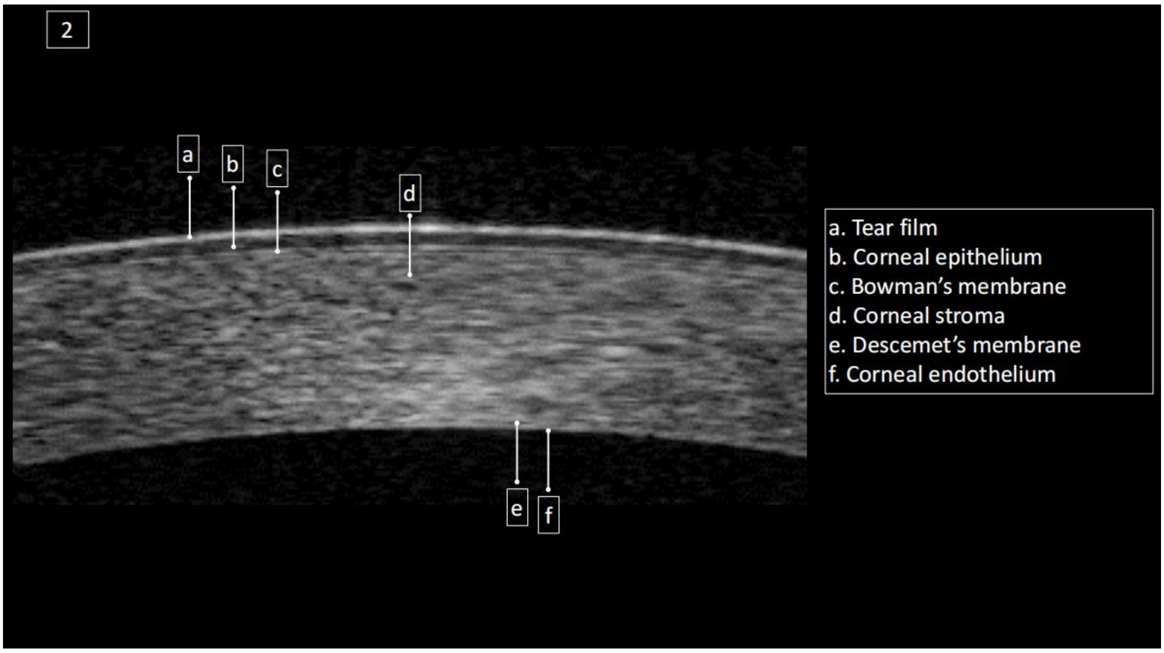

eFigure 2B. Unannotated Image

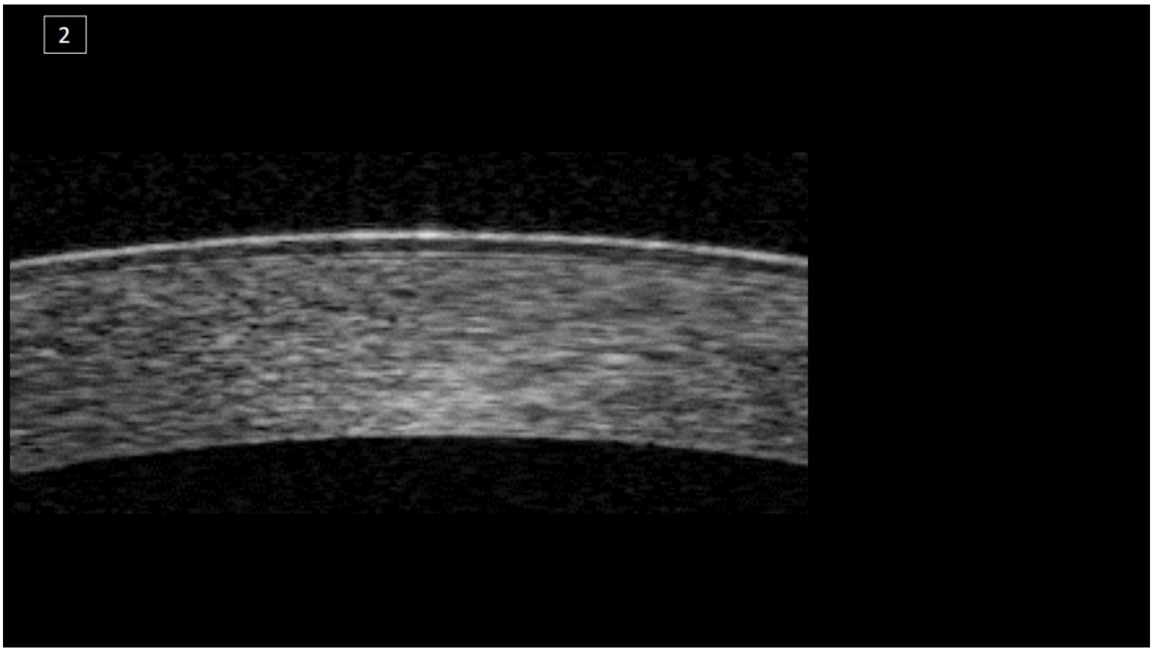

© 2025 Fraser AS et al. *JAMA Ophthalmology*.

eFigure 3. Limbal and corneoscleral labels presented to consensus group

Spectral domain OCT of 15-25 year old female acquired using Optovue Avanti

eFigure 3A. Annotated Image

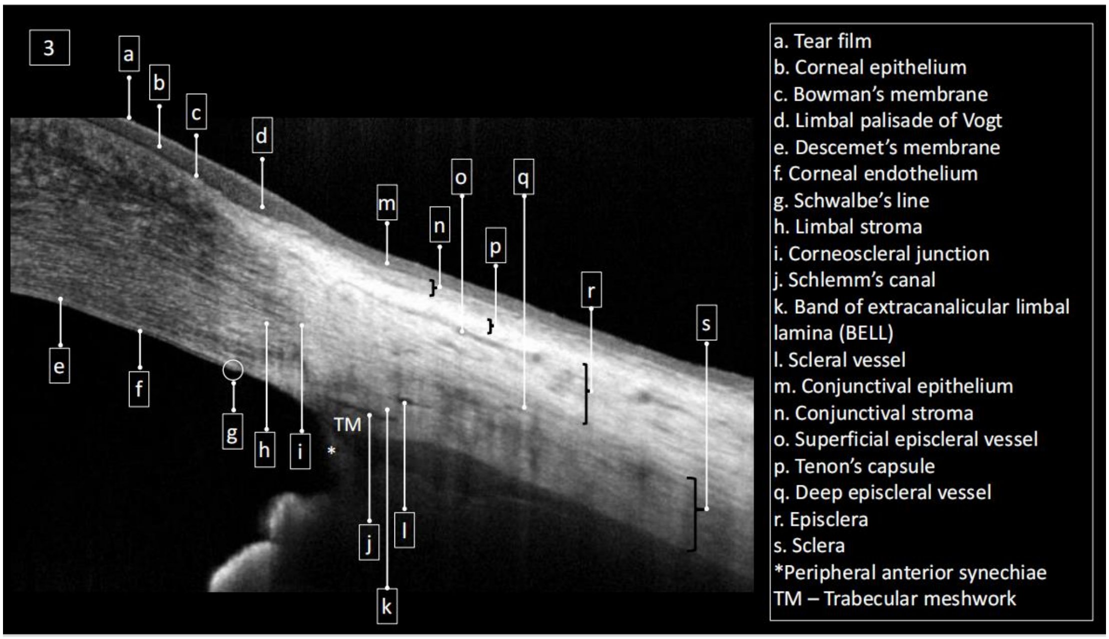

eFigure 3B. Unannotated Image

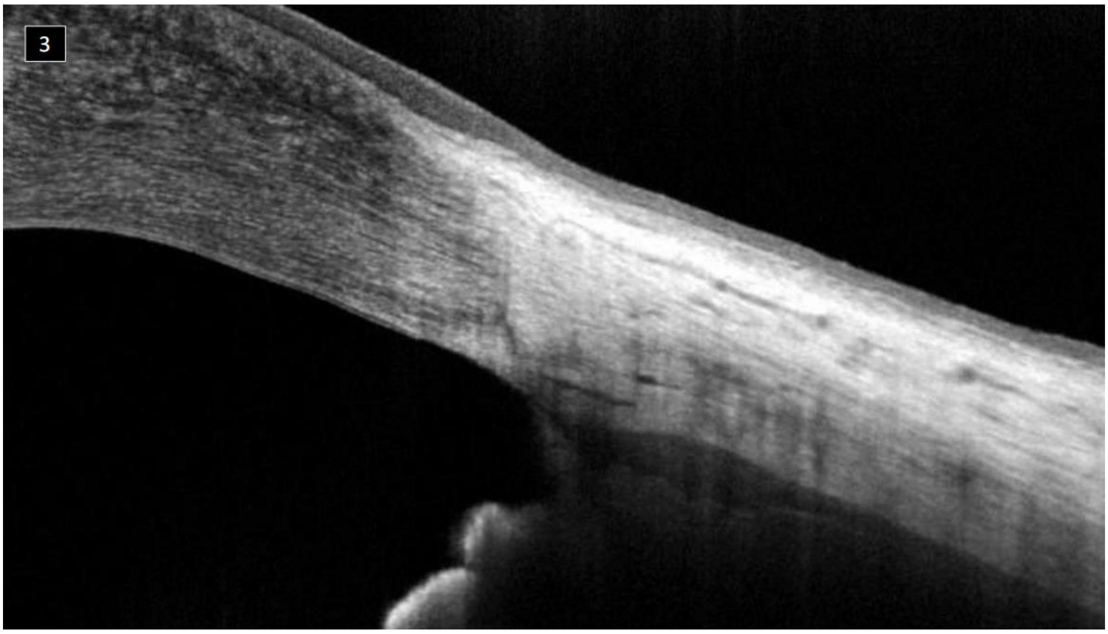

© 2025 Fraser AS et al. *JAMA Ophthalmology*.

eFigure 4. Limbal and corneoscleral labels presented to consensus group, part 2

Spectral domain OCT of 15-25 year old female acquired using Optovue Avanti

eFigure 4A. Annotated Image

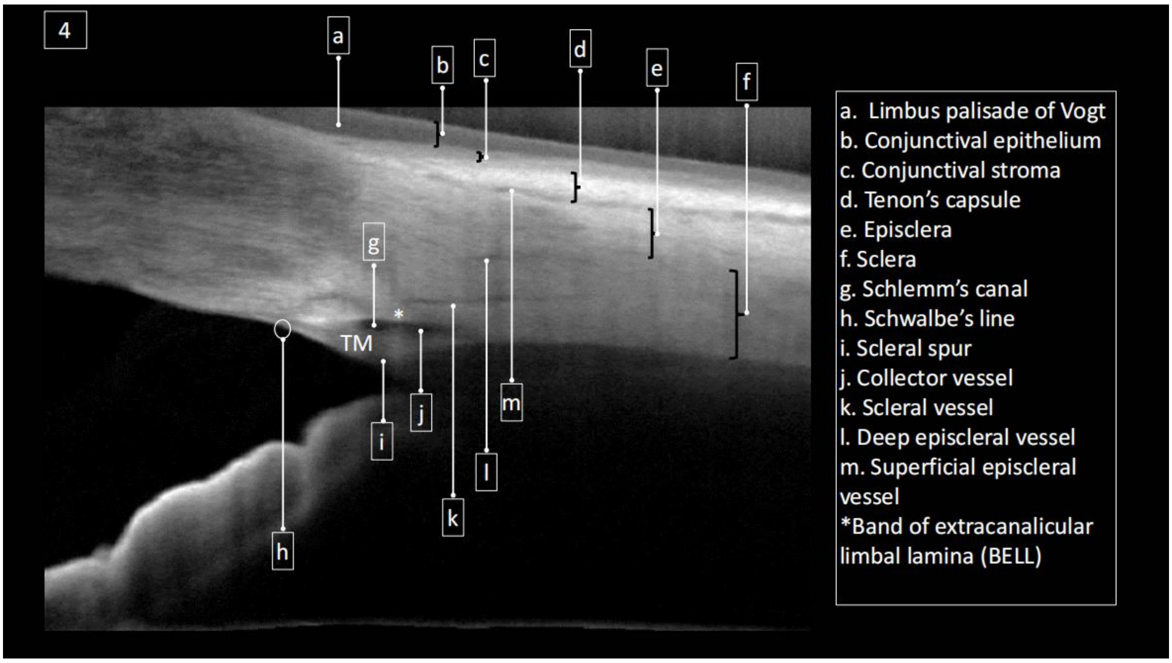

eFigure 4B. Unannotated Image

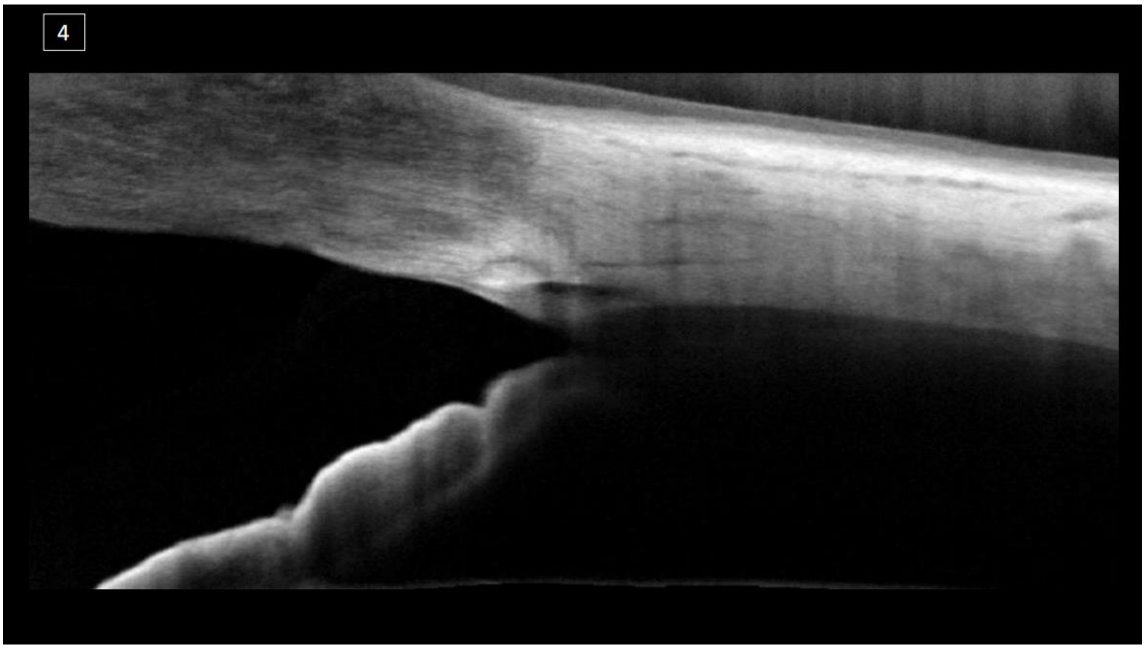

© 2025 Fraser AS et al. *JAMA Ophthalmology*.

eFigure 5. Limbal and anterior chamber angle labels presented to consensus group

Spectral domain OCT of 15-25 year old male acquired using Optovue Avanti.

eFigure 5A. Annotated Image

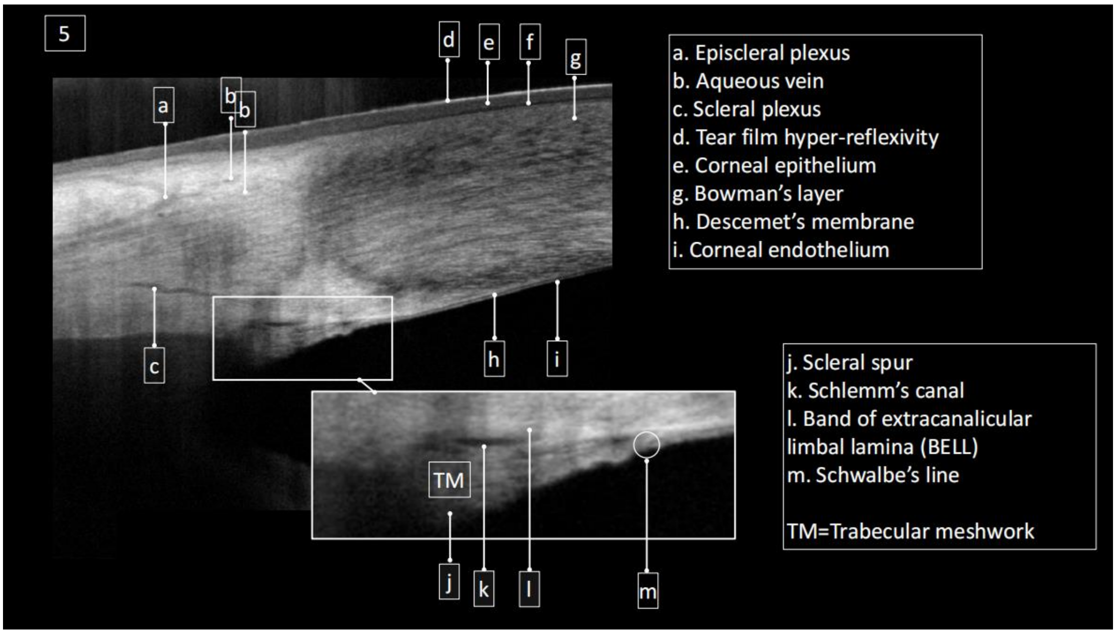

eFigure 5B. Unannotated Image

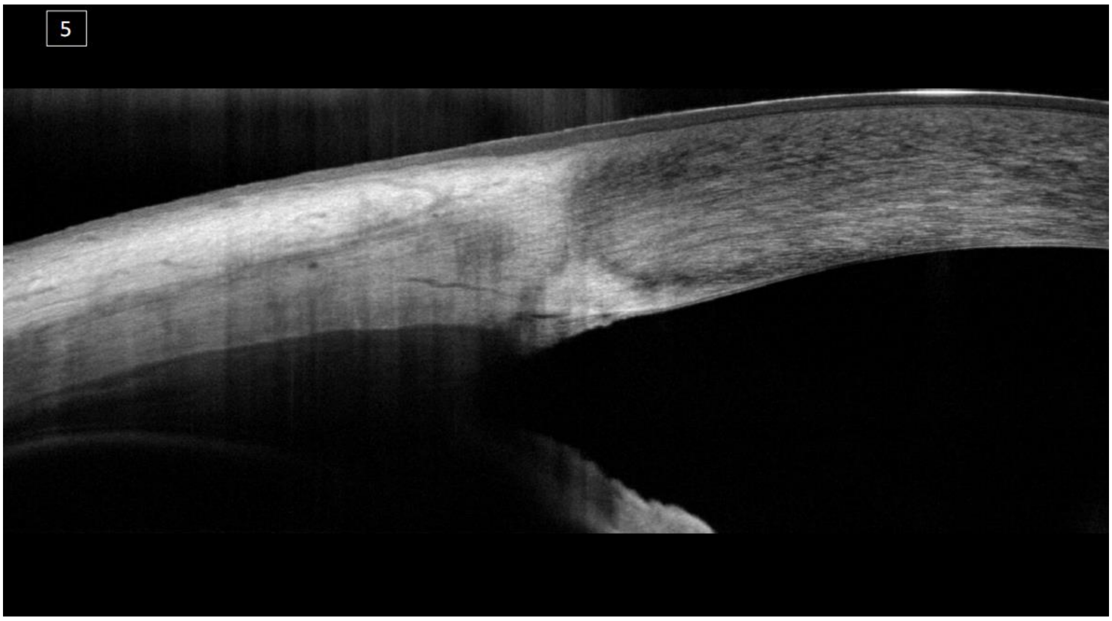

© 2025 Fraser AS et al. *JAMA Ophthalmology*.

eFigure 6. Crystalline lens structure labels presented to consensus group

Spectral domain OCT of 15-25 year old female acquired using Heidelberg Anterion

eFigure 6A. Annotated Image

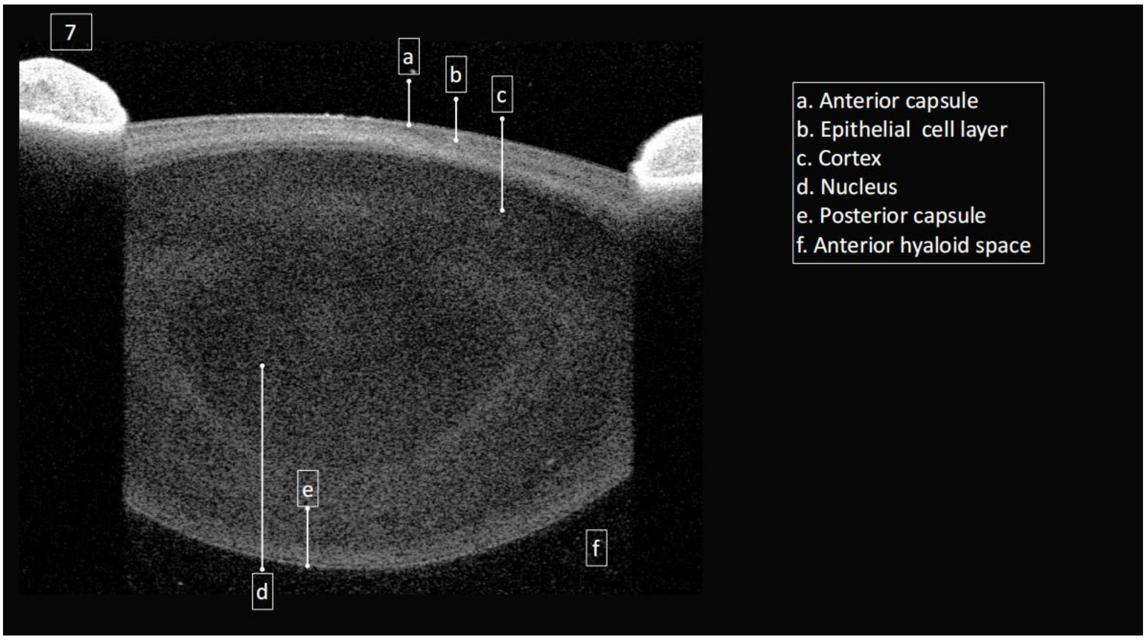

eFigure 6B. Unannotated Image

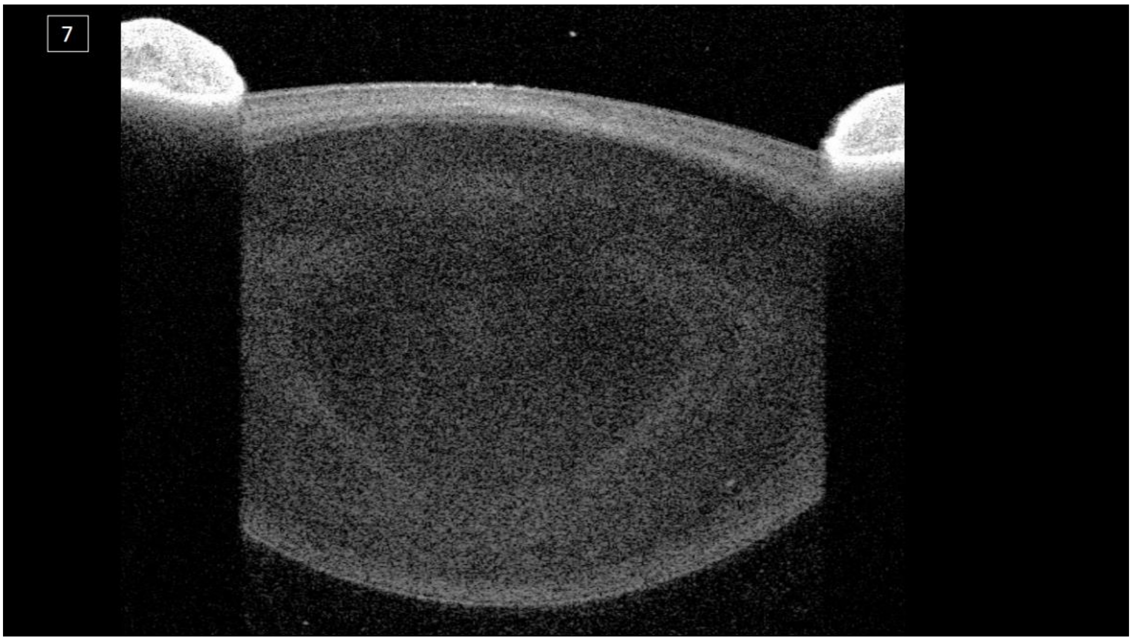

© 2025 Fraser AS et al. *JAMA Ophthalmology*.

eTable 2. Details of imaging systems used

Specifications as reported by manufacturer

**Name**	**Instrument type**	**A scan rate (Hz)**	**Wavelength nm**	**Optical resolution**
Optovue RTVue XR (Avanti)	Spectral domain	70,000	840	5 microns axially x 1.3 microns laterally
Heidelberg Anterion	Swept-source	50,000	1300	<10 microns axially × 30 microns laterally
Tomey CASIA2	Swept-source	50,000	1310	<10 microns axially × 30 microns laterally

© 2025 Fraser AS et al. *JAMA Ophthalmology*.
