# Supplementary material for: Proposed Nomenclature for Landmarks in Anterior-Segment OCT: The APOSTEL-AS Panel Consensus
Source: JAMA Ophthalmol. 2025 Aug 14;143(9):749–57. doi: 10.1001/jamaophthalmol.2025.2414 (PMC12355391; doi:10.1001/jamaophthalmol.2025.2414)
Supplement: Supplement 2. — Data Sharing Statement. [file jamaophthalmol-e252414-s002.pdf]

Data Sharing Statement

Fraser. Proposed Nomenclature for Landmarks in Anterior-Segment OCT. *JAMA Ophthalmol*. Published August 14, 2025. doi:10.1001/jamaophthalmol.2025.2414

Data
Data available: Yes
Data types: Data (not involving human participants)
How to access data: requests should be sent to a.solebo@ucl.ac.uk
When available: With publication

Supporting Documents
Document types: None

Additional Information
Who can access the data: To non-commercial researchers
Types of analyses: For research proposes
Mechanisms of data availability: With a signed data access agreement
